# Venous thromboembolic and hemorrhagic events after meningioma surgery: A single-center retrospective cohort study of risk factors

**DOI:** 10.1371/journal.pone.0273189

**Published:** 2022-08-16

**Authors:** Nebojsa Lasica, Djula Djilvesi, Vladimir Papic, Mladen Karan, Bojan Jelaca, Jagos Golubovic, Filip Pajicic, Milica Medic-Stojanoska, Petar Vulekovic, Lukas Rasulic

**Affiliations:** 1 Clinic of Neurosurgery, Clinical Center of Vojvodina, Novi Sad, Serbia; 2 Faculty of Medicine, University of Novi Sad, Novi Sad, Serbia; 3 Department of Neurosurgery, Haukeland University Hospital, Bergen, Norway; 4 Clinic for Endocrinology, Diabetes and Metabolic Diseases, Clinical Center of Vojvodina, Novi Sad, Serbia; 5 Faculty of Medicine, University of Belgrade, Belgrade, Serbia; 6 Clinic for Neurosurgery, Clinical Center of Serbia, Belgrade, Serbia; Universita degli Studi di Napoli Federico II, ITALY

## Abstract

Microsurgical resection of meningiomas in a majority of cases leads to a favorable outcome. Therefore, severe postoperative adverse events are less acceptable. The main purpose of this study was to investigate the incidence of symptomatic venous thromboembolism (VTE) and hemorrhagic complications in patients after operative treatment of intracranial meningiomas and to identify the risk factors in this patient subgroup. Of 106 patients undergoing elective craniotomy for meningioma overall incidence of symptomatic VTE was noted in 5.7% (six patients). For the risk-factor analysis older age (57.20 ± 11.60 vs. 71.00 ± 0.90 years, p < 0.001), higher body mass index (27.60 ± 4.80 vs. 33.16 ± 0.60 kg/m^2^, p < 0.001), WHO grade II (3.00% vs. 33.33%, p = 0.02), lower intraoperative blood loss (466.00 ± 383.70 vs. 216.70 ± 68.30 mL, p < 0.001), bedridden status and neurologic deficit (0.00% vs. 33.33%, p = 0.003 and 38.00% vs. 100.00%, p = 0.004) were associated with greater VTE risk. No risk factors for hemorrhagic complications were identified on univariate analysis. In conclusion, the incidence of VTE in meningioma patients is not negligible. Identified risk factors should be taken into account in the decision-making process for chemoprophylaxis when the risk of bleeding decreases.

## Introduction

The prevalence of pathologically confirmed meningiomas in the United States is approximately 97.5/100 000, with more than a twofold higher incidence among females [1]. Despite notable advances in modern treatment, open surgical removal remains the treatment of choice for the majority of patients [2]. Since meningiomas are usually benign lesions, and a favorable outcome is expected, severe postoperative complications are less acceptable [3]. Given that they are slowly growing and progress over several years, many patients that develop symptomatic and lesions that are large enough to require surgical treatment are older, and therefore, with many coexisting medical conditions [2].

Earlier studies suggested that patients with meningiomas carry a higher risk for thromboembolic complications when compared with other intracranial tumors, implying that risk may be tumor-specific [4,5]. Deep vein thrombosis (DVT), and pulmonary embolism (PE), known as venous thromboembolic (VTE) complications are considered severe, with an estimated mortality rate as high as 34% [6]. Increased risk for development of VTE events following resection of the brain tumors is assumed to occur due to hemostatic changes and hypercoagulable state caused by the tumor itself exacerbated by the patients’ initial non-ambulatory status during immediate post-surgical recovery [7]. Besides, the higher risk may also be attributable to longer surgical times associated with meningiomas [4].

Protocols for VTE prophylaxis are extensively discussed in neurosurgical literature; however, there are still disagreements and debates about the effectiveness and safety of the proposed strategies. Many neurosurgeons avoid the use of chemoprophylaxis with low molecular weight heparins (LMWH) postoperatively for fear of intracranial hemorrhage, while others think that the use of mechanical prophylaxis is not sufficient when used alone to prevent VTE [8].

In our study, we focused on patients undergoing elective craniotomy for microsurgical removal of intracranial meningiomas. The authors aimed to investigate the incidence and identify the risk factors in order to define high-risk patients for the development of VTE. To maximize study population homogeneity, we excluded patients with other preoperative treatment modalities, spinal meningiomas, meningiomatosis, due to potential influence on the analysis. Furthermore, we assessed the occurrence and factors that influence the rate of hemorrhagic complications to address the concerns brought about with the use of chemoprophylaxis.

## Materials and methods

### Patients and study design

**Fig 1:**
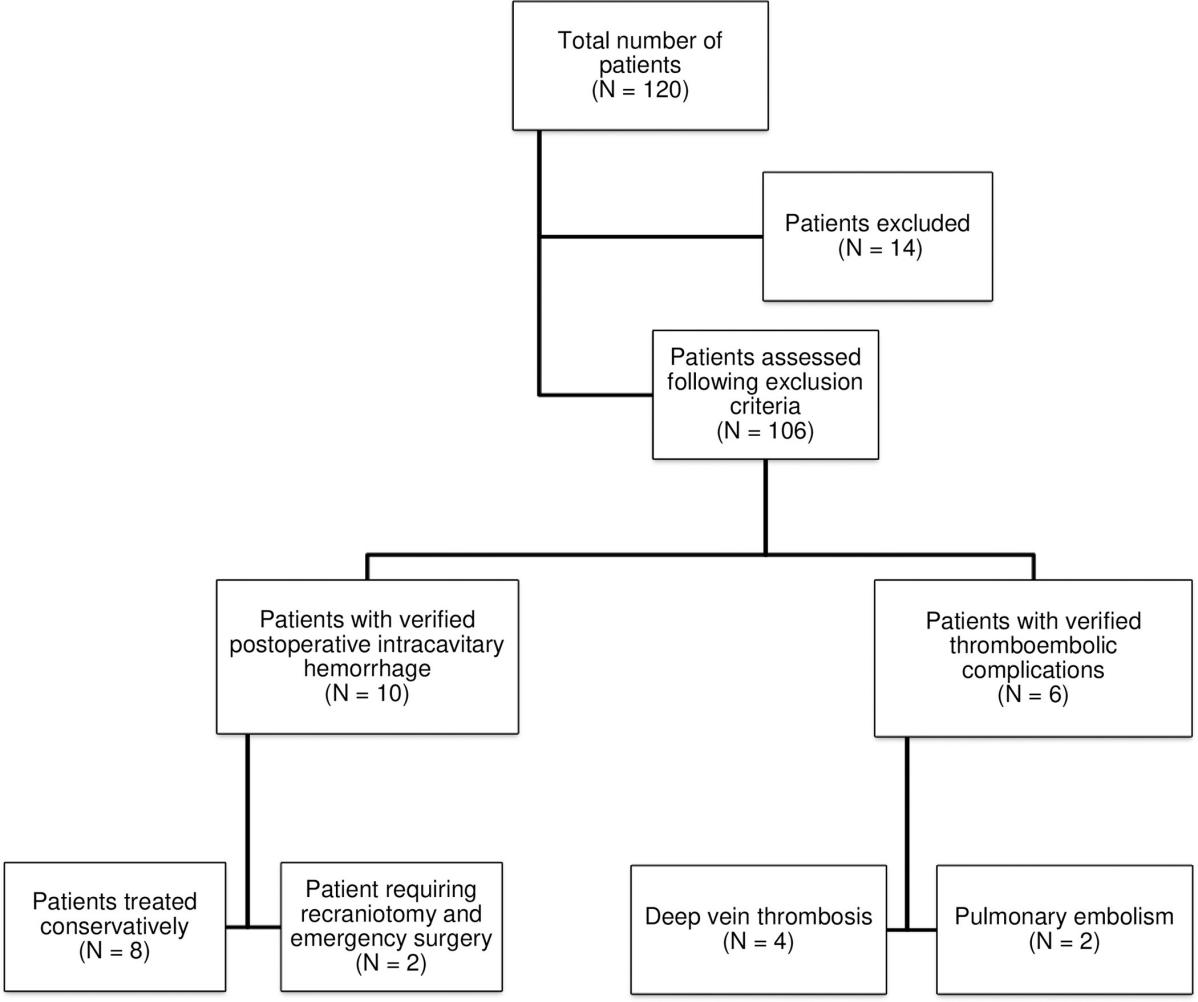
Flow chart of the postoperative hemorrhagic and thromboembolic complications in patients after operative treatment for intracranial meningiomas in our cohort.

A retrospective analysis of the hospital administrative database was performed, and all patients undergoing craniotomy for intracranial meningioma resection at our department between 2015 and 2019 were identified. The data was collected at the time of admission, surgery, and hospital discharge. The inclusion criteria for the study were 18 years of age and older, histologically verified meningioma, and a single meningioma. Patients who underwent biopsy, radiation therapy, and preoperative embolization, or transsphenoidal surgery, patients with spinal meningioma or meningiomatosis were excluded from this study to avoid possible effects on statistics. All procedures performed in studies involving human participants were in accordance with the ethical standards of the institutional and/or national research committee and with the 1964 Helsinki Declaration and its later amendments or comparable ethical standards. The study was approved by the institutional ethical review board (Ethical Review Board of the Clinical Center of Vojvodina number 00-15/340). Under our institutional ethical review board regulations, patient’s written informed consent was waived and was not required to collect and analyze the data. In Fig 1, study population analyzed is depicted.

### Variable definitions

The following data was extracted from the available electronic and paper medical records: age, gender, body mass index (BMI) and grade, AB0 blood type, presence of smoking habits, bleeding disorders and American Society of Anesthesiology (ASA) physical status classification system preoperatively; paretic limb and ambulatory status postoperatively; use of dexamethasone, use of pharmacologic VTE prophylaxis, preoperative use of antiplatelet agents/anticoagulants; confirmed intrahospital infection, presence of a central venous catheter, localization of the meningioma, operative duration, estimated intraoperative blood loss, and histologic grade and subtype. Patients’ ambulatory status was classified as ambulatory, walking aid, wheelchair-bound, and bedridden. Intracranial meningioma localizations were grouped into the following categories: falx/parasagittal, convexity, skull base, and posterior fossa.

### Body mass index classification

Patients were categorized according to the WHO classification into the following grades: underweight (<18.5 kg/m^2^), normal range (18.5–24.99 kg/m^2^), preobese (25–29.99 kg/m^2^), and obese (= 30 kg/m^2^) [9].

### Histological classification

The histologic grade and subtype of meningioma were extracted from the pathology report and assigned according to the 2016 World Health Organization (WHO) Classification of Tumors of the Central Nervous System [10].

### Postoperative adverse events

Postoperative adverse events, including DVT and PE, and postoperative intracranial hemorrhage during the hospital stay, were noted. Routine surveillance for VTE was not performed; instead, it was initiated in the case of clinical suspicion. The DVT was confirmed by duplex sonography of the leg vessels and PE by CT pulmonary angiography (CTPA). Postoperative hemorrhage was documented by reviewing the postoperative control head CT scan performed routinely on the first postoperative day. Additional head CT scans were performed as needed.

### VTE prophylactic regimen

In all patients included in this study, mechanical VTE prophylaxis was used routinely in the form of compression stockings. Patients received the pharmacologic VTE prophylaxis in the form of LMWH as needed (surgeons individual evaluation), receiving 2850 IU (0.3 mL) or 5700 IU (0.6 mL) (lower ambulatory status, increased weight, etc.) of nadroparin QD subcutaneously the day after the surgical treatment.

### Statistical analysis

The data obtained were analyzed using a commercially available statistical software package SPSS 21.0 for Windows (IBM Corp. in Armonk, NY). The univariate analysis of continuous variables was performed using a two-tailed independent sample T-test and reported as mean values ± standard deviations. Dichotomous variables were compared using the chi-squared test and Fisher’s exact test and presented as percentages within the group. All variables with a high probability of association (P-values <0.10) were considered for the final logistic regression. Odds ratio (OR), 95% confidence intervals (95% CI), and P-values are presented with postoperative VTE as outcome parameter. p-values <0.05 were considered significant. Our study was designed according to the STROBE Statement—a checklist of items for observational studies [11].

## Results

### Study population

During the inclusion period of four years, 120 patients who were operatively treated for meningioma at our department were enrolled in the study. In total, 106 patients met the aforementioned criteria and were included for further analysis (Fig 1).

**Fig 2:**
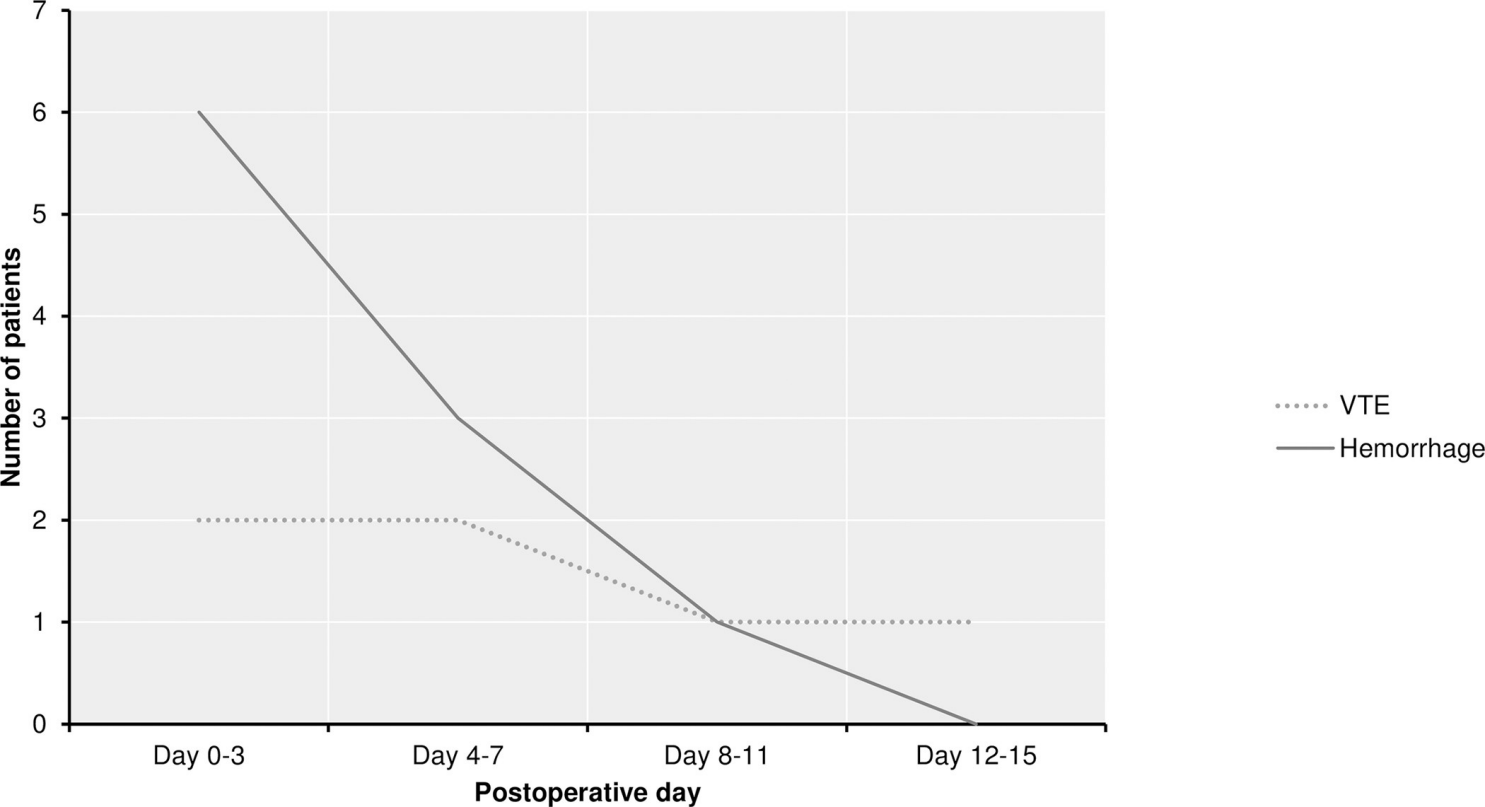
Timeline of postoperative thromboembolic and hemorrhagic complications. Most complications occur in the first postoperative week.

Of 106 patients included in our study, 78 were female and 28 male (female: male 40 ratio of 2.8: 1), with an average age of 58 years (range, 26 ± 78 years). VTE, in general, was recorded in six (5.7%) patients, of whom DVT occurred in four patients (3.8%) and PE in two patients (1.9%) during the hospital stay. In ten patients (9.4%), postoperative intracavitary hemorrhage was noted on the control head CT scan; however, only two were symptomatic and showed a mass effect on the control CT scan requiring surgical evacuation. The remaining eight patients with intracavitary blood with no compressive effect had no symptoms or neurological deficits. Therefore, the LMWH was initiated after the control head CT scan on the first postoperative day; additional CT scans performed revealed initial resorption of intracavitary blood that did not require a prolonged hospital stay. The majority of complications were observed during the first postoperative week, and there is a slight drop in the manifestation of VTE and hemorrhagic events during the second week. The adverse events timeline is shown in Fig 2 and is similar to previously reported results [3].

### Clinical characteristics of patients with and without postoperative VTE

The demographic and clinical characteristics of the VTE and non-VTE groups are summarized in Table 1.

**Table 1:** The univariate analysis of risk the factors for VTE complication in patients after operative treatment of intracranial meningiomas.

Variable	VTE (-) (N = 100)	VTE (+) (N = 6)	*p*-value
**Age (years)**	57.2 ± 11.6	71.0 ± 0.9	**<0.001**
**Gender**			
**Females (% within group)**	74.0	66.7	0.65
**Males (% within group)**	24.5	33.3	0.69
**BMI (kg/m** ^**2**^ **)**	27.6 ± 4.8	33.2 ± 0.6	**< 0.001**
**Histology**			
**WHO grade I (% within group)**	96.0	66.7	**0.04**
**WHO grade II (% within group)**	3.0	33.3	**0.02**
**WHO grade III (% within group)**	0.0	0.0	-
**Localization**			
**Convexity (% within group)**	18.0	0.0	0.60
**Falx/Parasagittal (% within group)**	28.0	66.7	0.07
**Skull base (% within group)**	34.0	33.3	1.00
**Posterior fossa (% within group)**	15.0	0.0	0.59
**ASA classification**			
**ASA II (% within group)**	2.0	0.0	0.73
**ASA III (% within group)**	97.0	100.0	0.67
**ASA IV (% within group)**	2.0	0.0	0.73
**Bleeding disorders**	2.0	0.0	0.73
**Central venous catheter (% within group)**	12.0	0.0	0.81
**Operating time (min)**	322.1 ± 131.4	238.3 ± 75.7	0.13
**Estimated intraoperative blood loss (mL)**	466.0 ± 383.7	216.7 ± 68.3	**< 0.001**
**Bedridden status (% within group)**	0.0	33.3	**0.003**
**Paretic limb (% within group)**	38.0	100.0	**0.004**
**Intrahospital infection (% within group)**	8.0	33.3	0.09
**Dexamethasone use (days)**	15.6 ± 6.9	15.3 ± 1.9	0.83
**LMWH use (% within group)**	68.0	100.0	0.17
**Smoking habits (% within group)**	34.0	66.7	0.18
**Non-0 blood type (% within group)**	54.0	66.7	0.69

ASA—American Society of Anesthesiology. All bold values are significant with a *p*-value < 0.05.

**Fig 3:**
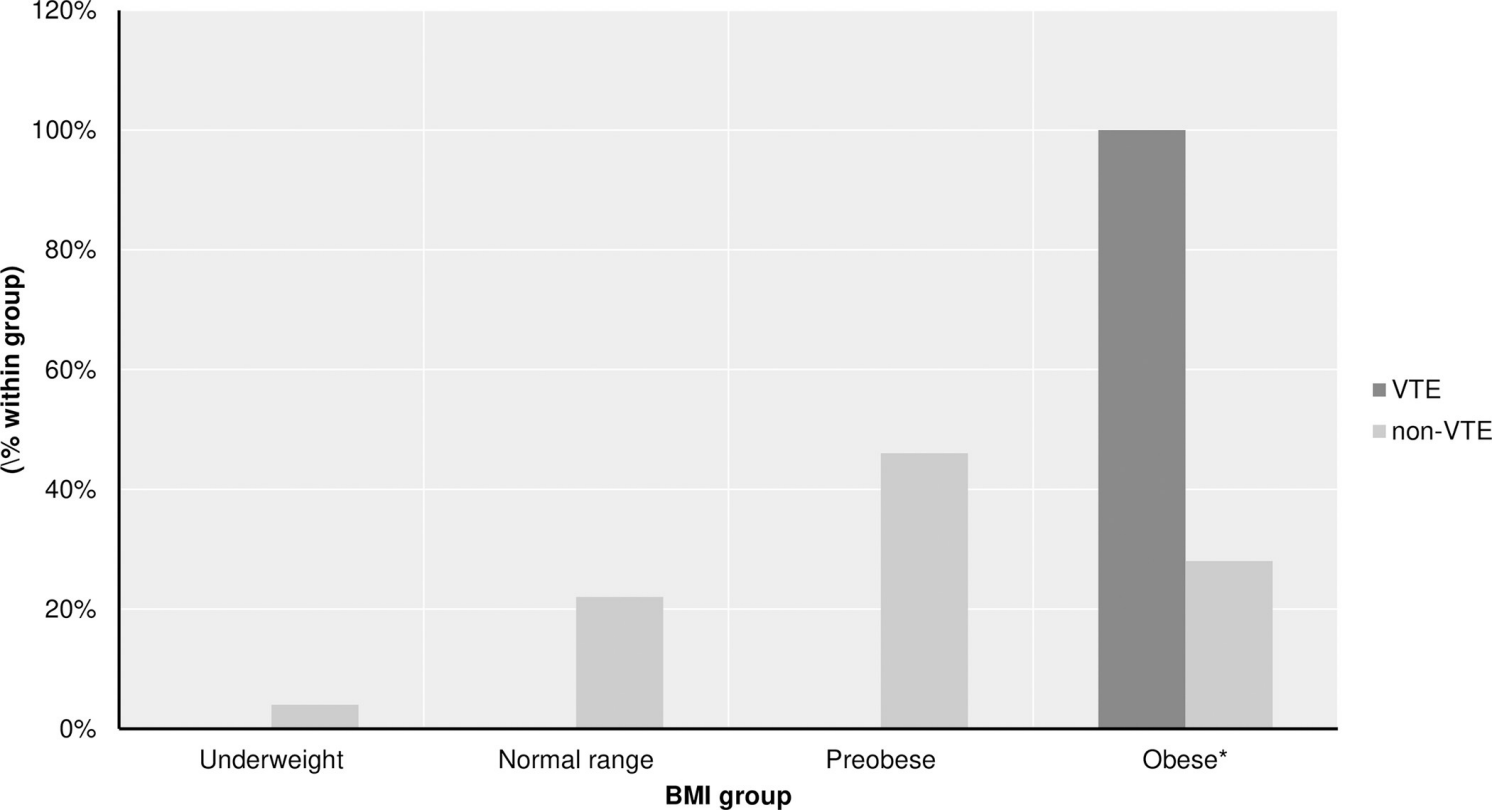
Between-group comparison of BMI grade of patients from the VTE and the Non-VTE group. Patients with VTE are exclusively present in the obese group. *Values are significant with a p-value <0.05.

There was balanced distribution regarding days of dexamethasone use and skull base meningiomas in both groups. On average, patients within the VTE group were significantly older than non-VTE. When comparing BMI measured before surgery, patients from the VTE group showed higher BMI than the non-VTE group, and more frequently belonged to the obese group (Fig 3).

**Fig 4:**
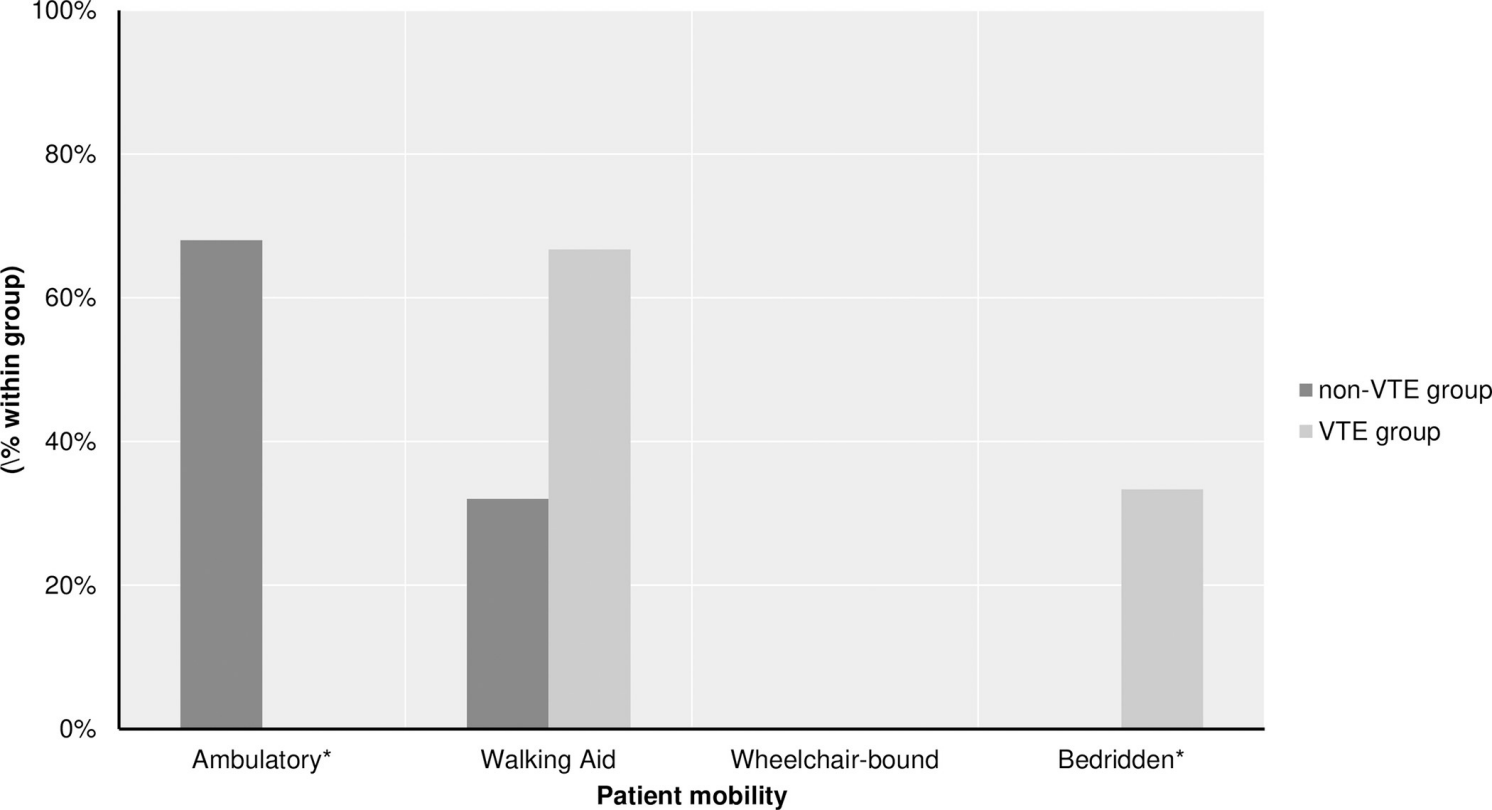
Between-group comparison of ambulatory status of patients in the VTE and Non-VTE group. VTE group patients had exclusively bedridden status, and non-VTE patients were all ambulatory. *Values are significant with a p-value <0.05.

VTE group contained less WHO grade I and more WHO grade II meningiomas, and when comparing histological subtypes, atypical meningiomas were more frequent in the VTE group (4.0% vs. 33.3%, p = 0.04) (S1 Table). There was less intraoperative loss observed and shorter procedure time in the VTE group than the non-VTE group. Bedridden status in the postoperative course was witnessed in VTE patients more often, together with the presence of paretic limb; on the other hand, patients in the non-VTE group were all ambulatory in the postoperative course (68.0% vs. 0.00%, p = 0.003) (Fig 4).

It is important to note that paresis was consistently present in the same limb as the DVT. Female gender, convexity, and posterior fossa meningiomas, the presence of a central venous catheter, and bleeding disorders were less frequent in the VTE group, and smoking and non-0 blood type were more frequent; however, with no observed significance. No difference was observed in ASA categories between VTA and non-VTE groups. All patients in the VTE group received LMWH, however, when compared with the non-VTE group, with no statistical significance. Ultimately, according to previously established criteria, age, BMI, histologic grade, estimated blood loss, bedridden status, the presence of the paretic limb, confirmed intrahospital infection, and falx/parasagittal localization were used as variables for the final binary logistic regression.

### Logistic regression analysis of selected risk factors

Age (OR 1.280, 95% CI 1.049–1.561, p = 0.015) and BMI (OR 1.302, 95% CI 1.063–1.594, p = 0.011) significantly influenced the occurrence of VTE; however, WHO grade, estimated blood loss, bedridden status, the presence of the paretic limb, confirmed intrahospital infection, and falx/parasagittal localization of meningioma did not show significant influence on the occurrence of the VTE complications.

### Clinical characteristics of patients with and without postoperative hemorrhage

According to patient chart data, the pharmacologic VTE prophylaxis was used in 74 88 (69.8%) patients, and a history of preoperative use of antiplatelet agents/anticoagulants was noted in 16 (15.1%) patients. Table 2 summarizes the results of the univariate comparison of risk factors for postoperative hemorrhage in meningioma patients.

**Table 2:** Analysis of risk factors for postoperative hemorrhage in patients after operative treatment of intracranial meningiomas.

Variable	IH (-) (N = 96)	IH (+) (N = 10)	*p*-value
**Age (years)**	57.9 ± 12.0	58.2 ± 9.5	0.95
**Gender (female, % within group)**			
**Females (% within group)**	70.8	100.0	0.11
**Males (% within group)**	29.2	0.0	0.1
**BMI (kg/m** ^**2**^ **)**	27.8 ± 4.9	28.7 ± 3.5	0.66
**Operating time (min)**	316.1 ± 125.0	329.2 ± 184.5	0.83
**Estimated blood loss (mL)**	416.7 ± 319.3	790.0 ± 667.0	0.11
**Dexamethasone use (days)**	15.5 ± 6.8	15.6 ± 5.7	0.98
**Preexisting use of antiplatelet agents/anticoagulation (% within group)**	16.7	0.0	0.35
**Use of postoperative pharmacologic VTE prophylaxis (% within group)**	70.8	60.0	0.48
**Histology**			
**WHO grade I (% within group)**	93.8	100.0	0.93
**WHO grade II (% within group)**	5.2	0.0	1.00
**WHO grade III (% within group)**	0.0	0.0	-

IH–intracavitary hemorrhage.

There was an evenhanded distribution considering age, BMI, operating time, and the days of dexamethasone use. Only females were present in the group with postoperative hemorrhage; however, the between-group comparison failed to show statistical significance. There was greater estimated blood loss observed in the group with the documented postoperative hemorrhage, yet, with no significance. The use of antiplatelet agents or anticoagulation before surgery was not observed in this study’s postoperative hemorrhage group. Patients within the group with confirmed postoperative hemorrhage less frequently received LWMH prophylaxis for VTE. WHO grade I was exclusively present in a group with postoperative hemorrhage; however, no difference in histological subtype was observed between groups (S2 Table). As none of the risk factors considered in univariate analysis reached the previously established p-value of 0.1, no further logistic regression no further logistic regression was performed.

## Discussion

VTE is a significant complication among neurosurgical patients, given that it increases morbidity and mortality and leads to a more prolonged stay with increased hospital charges [12]. An early study with I^125^-labeled fibrinogen leg scans has shown that the rate of deep vein thrombosis (DVT) in patients with meningioma can go as high as 72%, a much higher rate than in glioblastomas (60%) and brain metastases (20%) [13]. Despite the evidence of a higher frequency, it is still widely speculated about the possible underlying pathophysiological mechanisms that lead to VTE in patients with intracranial meningiomas. On the other hand, an attempt to prevent postoperative thromboembolic complications is often accompanied by indecisiveness since neurosurgeons avoid reaching for the pharmacologic VTE prophylaxis lightly in the immediate postoperative period out of fear of severe hemorrhagic complications [14]. This study’s primary goal was to assess the incidence and to identify the patients and their characteristics accompanied by a higher risk of VTE.

Among demographical variables, the risk for developing venous thromboembolism was shown to be strongly age-related, with an increase in the incidence and prevalence for both men and women, with a reported increase of almost 90 fold from <15 to >80 years of age [15–17]. In patients with intracranial meningiomas, age has been repetitively identified as an independent risk factor for VTE [12,18–21]. Aging is accompanied by changes that lead to a higher risk of developing thrombotic events in the elderly that involve various hemostatic system components. Advanced age leads to a gradual increase in plasma concentrations of some coagulation factors as well as enhanced activity of the coagulation enzymes [22–25]; there is also a documented decline in fibrinolytic activity [26], increased platelet activation [27,28] and alterations in vascular endothelium function, and rise in vascular wall’s stiffness due to calcium and collagen deposition [29]. In this study, the mean age in the group with DVT and/or PE was significantly higher than in the group with no thromboembolic events observed (71.0 ± 0.9 vs. 57.2 ± 11.6 years, p < 0.05), which coincides with previously reported results for neurosurgical patients undergoing craniotomy [20,30,31].

The influence of obesity on overall health and various diseases is well established, particularly on thrombotic disorders, including cardiovascular disease, stroke, and venous thromboembolism [32]. According to the International Society on Thrombosis and Haemostasis categorization of venous thromboembolism, obesity belongs to the group of weak but both transient and persistent risk factors for developing VTE [33,34]. Many pathways could contribute to obesity-related thrombosis, including chronic inflammation mediated by cytokine secreting adipocytes, increased intra-abdominal and femoral vein pressure, and venous stasis [35,36]. This study showed evidence that higher BMI influenced the rate of VTE events in patients with meningioma (27.6 kg/m^2^ vs. 33.2 kg/m^2^, p < 0.001), in this case also confirming results from previous studies [20,37].

Even though our final logistic regression failed to correlate the influence of higher histologic grade, estimated intraoperative blood loss, postoperative bedridden status, and the presence of the paretic limb, it is essential to point out that there was a statistical difference between VTE and the non-VTE group observed. Active cancer has been recognized as a strong risk factor for VTE complications [33,34]. Various mechanisms may play a role, including tumor-induced hypercoagulative state as well as increased adhesion and platelet aggregation [38]. Previous studies report conflicting results on correlation between WHO grade and incidence of VTE [20,37,39]. However, in total, only five patients were diagnosed with atypical meningioma (WHO grade II) in our cohort, and none with malignant meningioma (WHO grade III) that may prove insufficient in drawing conclusions regarding higher grade and incidence of VTE complications; therefore, further research and larger cohort may be necessary for unambiguous conclusions. It is uncertain what could be a possible explanation of lower incidence of postoperative thromboembolic events in patients with higher intraoperative estimated blood loss; however, it is reasonable to presume that hemodilution might play a protective role against VTE, as suggested previously [40]. The presence of paretic limb and bedridden status of the patient, on the other hand, could contribute to VTE complications via stasis due to the impeded venous return of the blood [41], and subsequently, attending neurosurgeon should always advocate early mobilization of patients as one of the most valuable modalities of thromboprophylaxis as demonstrated in this study. Both prolonged immobility and focal neurological deficit with leg paresis are recognized risk factors in previous studies and considered to be in strong correlation with VTE complications [17,33,34].

Our results imply that none of the evaluated risk factors in this study contributed to hemorrhagic complications in elective meningioma surgery. Therefore, it should not play a significant role in the decision-making process when considering pharmacologic VTE prophylaxis. There is a lack of high-level evidence in the literature regarding the timing and modality of VTE prophylaxis, and recommendations are primarily based on expert opinion and are, therefore, highly variable [14]. Current standards recommend VTE prophylaxis based on a type of underlying pathology, and at minimum, use of mechanical prophylaxis, preferably with intermittent pneumatic compression (IPC) device for patients requiring craniotomy [42]. Therefore, pharmacologic VTE prophylaxis should perhaps be reserved for carefully selected patients, i.e., the ones with several risk factors for the development of VTE and the ones where the benefit would exceed the risk of bleeding.

We acknowledge that this study is retrospective, and despite the significant effort in medical record examinations, limitations are a result of the retrospective manner of data collecting. Despite thorough analysis, there is a possibility that our study underestimates the rate of complications. Lack of prospective screening for DVT and PE that may be clinically silent, it is likely that the full extent of thromboembolic adverse event is underestimated due to unintentional exclusion of asymptomatic patients.

## Conclusions

Despite the aforementioned limitations of our retrospective approach, we provide evidence that higher body mass index, older age, WHO grade II meningiomas, lower intraoperative blood loss, bedridden status, and the presence of neurologic deficit was accompanied by an increased rate of VTE complications in patients undergoing elective craniotomy for meningioma surgery and therefore, should play a role in the decision-making process when considering VTE prophylaxis. The use of pharmacologic VTE prophylaxis should always be considered in patients with a higher risk of developing VTE events when the risk of bleeding decreases.

## Supporting information

S1 Table The Univariate Analysis of WHO histological subtype in groups with and without VTE. (DOCX)

S2 Table WHO histological subtype in groups with and without intracavitary hemorrhage. (DOCX)

S1 Data (SAV)
