# Supplementary material for: Venous thromboembolic and hemorrhagic events after meningioma surgery: A single-center retrospective cohort study of risk factors
Source: PLoS One. 2022 Aug 16;17(8):e0273189. doi: 10.1371/journal.pone.0273189 (PMC9380925; doi:10.1371/journal.pone.0273189)
Supplement: S1 Table — (DOCX) [file pone.0273189.s001.docx]

**Supplementary Table 1.**  Univariate analysis of WHO histological subtypes in the groups with and without VTE.

| **WHO grade** | **VTE (-) (N = 100)** | **VTE (+) (N = 6)** | ***p*-value** |
| --- | --- | --- | --- |
| **WHO grade I** | | | |
| **Meningothelial meningioma: N (% within group)** | 2 (2.00) | 0 (0.00) | 0.63 |
| **Fibrous meningioma: N (% within group)** | 32 (32.00) | 2 (33.3) | 1.00 |
| **Transitional meningioma: N (% within group)** | 56 (56.00) | 2 (33.33) | 0.51 |
| **Psammomatous meningioma: N (% within group)** | 4 (4.00) | 0 (0.0) | 1.00 |
| **Angiomatous meningioma: N (% within group)** | 2 (2.00) | 0 (0.00) | 1.00 |
| **Microcystic meningioma: N (% within group)** | - | - | - |
| **Secretory meningioma: N (% within group)** | - | - | - |
| **Lymphoplasmacyte-rich meningioma: N (% within group)** | - | - | - |
| **Metaplastic meningioma: N (% within group)** | - | - | - |
| **WHO grade II** | | | |
| **Chordoid meningioma: N (% within group)** | - | - | **-** |
| **Clear cell meningioma: N (% within group)** | - | - | **-** |
| **Atypical meningioma: N (% within group)** | 4 (4.00) | 2 (33.33) | **0.04** |
| **WHO grade III** | | | |
| **Papillary meningioma: N (% within group)** | - | - | - |
| **Rhabdoid meningioma: N (% within group)** | - | - | - |
| **Anaplastic (malignant) meningioma: N (% within group)** | - | - | - |

All bold values are significant with p-value < 0.05
